# Effect of Spontaneous Subarachnoid Hemorrhage on Cerebrospinal Fluid Indicators

**DOI:** 10.3390/brainsci13050778

**Published:** 2023-05-10

**Authors:** Huichao You, Wenqi Li, Qianxue Chen

**Affiliations:** 1 Department of Neurosurgery, Renmin Hospital of Wuhan University, Wuhan 430060, China; youhuichao1115@163.com; 2 Department of Ultrasound, Hubei No. 3 People’s Hospital of Jianghan University, Wuhan 430033, China

**Keywords:** subarachnoid hemorrhage, intracranial infection, cerebrospinal fluid routine, cerebrospinal fluid biochemistry, leukocyte

## Abstract

The cerebrospinal fluid (CSF) analysis in ruptured aneurysms can be greatly affected by subarachnoid hemorrhage (SAH), making the diagnosis of intracranial infection more difficult after surgery. This study aimed to identify the reference value range of CSF in the pathological state following spontaneous SAH. A retrospective analysis of demographic and CSF data of all spontaneous SAH patients treated between January 2018 and January 2023 was conducted. A total of 101 valid CSF specimen data were collected for analysis. Our results indicate that in 95% of patients after spontaneous SAH, the leukocyte count in CSF was less than 880 × 10^6^/L. Additionally, the proportion of neutrophils, lymphocytes, and monocytes did not exceed 75%, 75%, and 15%, respectively, in 95% of the population. Furthermore, in 95% of the specimens, the concentration of chloride, glucose, and protein was >115 mmol/L, >2.2 mmol/L, and <2.3 g/L, respectively. Compared to the normal reference values, the CSF indexes after spontaneous SAH showed significant changes, especially in the leukocyte count, chloride concentration, and glucose concentration. Using “white blood cell count < 880/mm^3^, glucose > 2.2 mmol/L, chloride > 115” as the reference values for SAH pathological status is more meaningful for reference purposes.

## 1. Introduction

Cerebrospinal fluid (CSF) laboratory testing is necessary to aid in the diagnosis of infection. However, the number of CSF cells and range of biochemical indicators in patients with subarachnoid hemorrhage (SAH) can change significantly due to bloody CSF, making the diagnosis of intracranial infection more challenging. To date, no studies have been conducted to determine the fluctuation range of CSF indicators in the pathological state following spontaneous SAH. In theory, the probability of intracranial infection caused by intravascular intervention treatment for aneurysms is extremely low because it does not result in a breach of the blood-brain barrier. This study collected routine and biochemical results of CSF from SAH patients who received interventional embolization or conservative treatment and analyzed the fluctuation interval to identify the reference value range of CSF in the pathological state following spontaneous SAH.

## 2. Materials and Methods 

### 2.1. Study Design and Participants

Cases of spontaneous SAH were continuously enrolled at the Third People’s Hospital of Hubei Province from January 2018 to January 2023. Patients or their legal representatives signed both the informed consent form for diagnosis and treatment, as well as the informed consent form for this study. The inclusion criteria were as follows: (1) patients with spontaneous SAH; (2) patients who underwent interventional therapy or conservative treatment after admission; (3) patients without pre-existing nervous system diseases prior to hospitalization; and (4) patients who underwent lumbar puncture after SAH. The exclusion criteria comprised the following: (1) Patients who underwent craniotomy after admission; (2) patients who underwent other invasive neurological procedures besides aneurysm embolization prior to CSF collection; (3) patients whose CSF culture indicated bacterial infection; and (4) patients who experienced increased SAH during or after interventional therapy. All procedures were conducted in compliance with relevant guidelines and regulations. This study received approval from the local ethics committee.

### 2.2. Procedure

A retrospective study was conducted to collect all cases of spontaneous SAH from January 2018 to January 2023. Patient data, including age, gender, time of SAH, time of lumbar puncture, and various CSF indicator results, were recorded. Cases were screened according to inclusion and exclusion criteria, and data from all eligible cases were analyzed.

### 2.3. Statistical Analysis

Statistical analyses were conducted using IBM SPSS Statistics version 26 (IBM Corp., Armonk, NY, USA). The normality of the measurement data was tested. Continuous variables were presented as medians with interquartile ranges (IQR) and compared using the Mann-Whitney U test. Categorical variables were reported as numbers with percentages. *p* < 0.05 was considered statistically significant.

## 3. Results

### 3.1. Demographic and Clinical Data

A total of 118 cases of spontaneous SAH treated with either interventional or conservative methods were included in this study. After exclusion criteria were applied, a total of 101 eligible CSF specimens were collected. Of these, 86 cases (85.1%) underwent endovascular embolization, while 15 cases (14.9%) were treated conservatively (Table 1). The primary reason for choosing conservative treatment was the absence of vascular lesions related to SAH, including intracranial aneurysms. The median age of the patients was 54 years (range, 28–79), with 34 of them being male (33.7%). The CSF was collected at a median of 6 days (range 1–15) after the onset of bleeding.

### 3.2. Characteristics of Routine Examination of CSF

**Figure 1:**
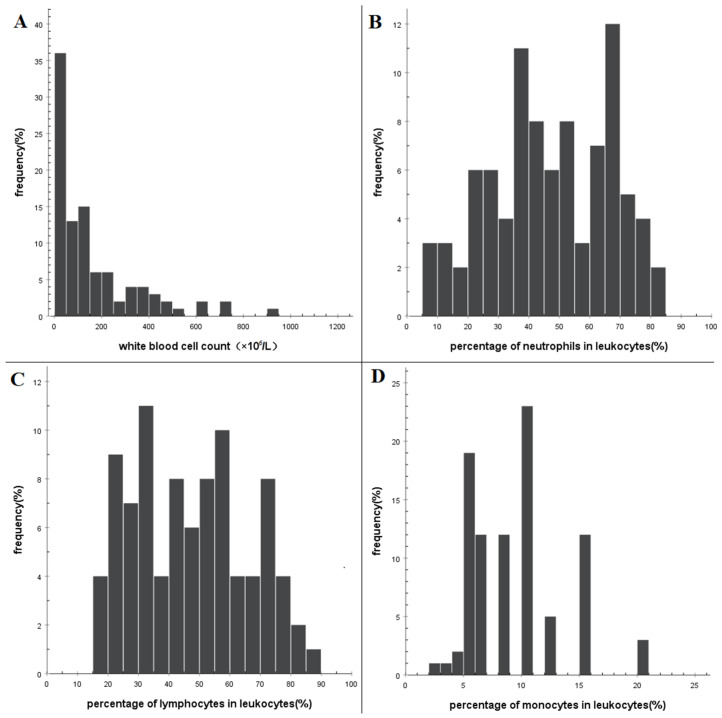
Histogram showing the distribution of leukocyte counts in cerebrospinal fluid after spontaneous subarachnoid hemorrhage. (**A**) Frequency distribution of leukocyte counts, frequency distribution of the proportion of (**B**) neutrophils, (**C**) lymphocytes, and (**D**) monocytes.

In patients with spontaneous SAH, the white blood cell count (WBC, leukocyte) of all specimens showed a skewed distribution (Figure 1A). The median was 100 × 10^6^ cells/L, the 5–95th percentiles were (8–880) × 10^6^ cells/L, and the 10–90th percentiles were (10–496) × 10^6^ cells/L (Table 2). In terms of the comparison of WBC counts in CSF among different groups, the male group had lower counts than the female group, and the conservative treatment group had lower counts than the interventional group. The differences were statistically significant (Table 1). The proportion of CSF leukocyte classification exhibited a skewed distribution (Figure 1B–D). The median proportion of neutrophils was 46.0%, with 5th–95th percentiles ranging from 10% to 75% and 10t–90th percentiles ranging from 20% to 70% (Table 2). The proportion of lymphocytes in CSF had a median of 45.0%, with 5–95th percentiles ranging from 18.2% to 75.0% and 10–90th percentiles ranging from 20.0% to 73.8% (Table 2). Similarly, the median proportion of monocytes in CSF was 8.0%, with 5th–95th percentiles ranging from 4.6% to 15.0% and 10–90th percentiles ranging from 5.0% to 10.0% (Table 2). After spontaneous SAH, the white blood cells in the cerebrospinal fluid did not exceed 500 × 10^6^/L in 90% of patients and were less than 880 × 10^6^/L in 95% of patients. The proportion of neutrophils, lymphocytes, and monocytes in 95% of specimens did not exceed 75%, 75%, and 15%, respectively. 

**Figure 2:**
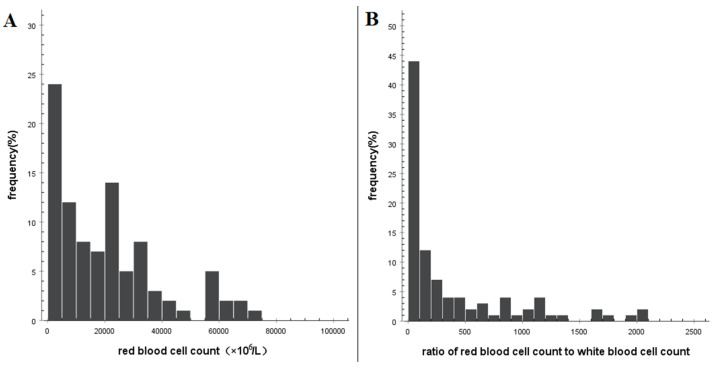
Histogram showing the distribution of erythrocyte counts in cerebrospinal fluid after spontaneous subarachnoid hemorrhage. (**A**) Frequency distribution of erythrocyte counts. (**B**) Frequency distribution of the ratio of erythrocyte counts to leukocyte counts.

The red blood cell (RBC or erythrocyte) count in CSF increased and exhibited a skewed distribution (Figure 2A). The median RBC count was 19,000 × 10^6^ cells/L, with 5–95th percentiles ranging from (290–355,642) × 10^6^ cells/L and 10–90th percentiles ranging from (900–66,162) × 10^6^ cells/L (Table 2). The ratio of RBC count to WBC counts in CSF after SAH was also skewed (Figure 2B). The median ratio was 122.6, with 5–95th percentiles ranging from 5 to 2478 and 10–90th percentiles ranging from 9 to 1670 (Table 2). These findings indicate that the ratio of RBC count to WBC count in CSF exhibits considerable variation among patients following subarachnoid hemorrhage.

### 3.3. Characteristics of Biochemical Examination of CSF

**Figure 3:**
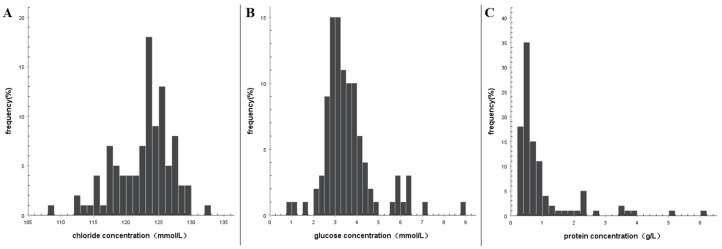
Histograms showing the distribution of fluctuations in biochemical indices of cerebrospinal fluid after spontaneous subarachnoid hemorrhage. (**A**) Chloride concentration, (**B**) glucose concentration, and (**C**) protein concentration.

The distribution of chloride concentration was found to be skewed (Figure 3A), with the 5–95th percentiles range of (114.9–128.9) mmol/L and the 10–90th percentiles range of (116.8–127.2) mmol/L. The median chloride concentration was 123.0 mmol/L (Table 2). It is noteworthy that in 95% of the specimens, the chloride concentration exceeded 115 mmol/L. The comparison of chloride concentration in CSF among different groups showed significant statistical differences (Table 1). The concentration in the elderly group was lower than that in the non-elderly group, while the concentration in the group with CSF collection time exceeding one week was lower than that in the other groups. The glucose concentration distribution was found to be skewed (Figure 3B), with the 5% to 95% percentile range of (2.2–6.3) mmol/L and the 10% to 90% percentile range of (2.6–5.5) mmol/L and the median of 3.3 mmol/L (Table 2). In 95% of the samples, the glucose concentration was greater than 2.2 mmol/L. When comparing different groups, the interventional embolization group had significantly lower glucose concentrations than the conservative group, with statistical significance (Table 1). The distribution of protein concentration was skewed (Figure 3C), with the 5–95th percentiles range of (0.2–3.5) g/L and the 10–90th percentiles range of (0.3–2.3) g/L. The median protein concentration was 0.6 g/L (Table 1). In 95% of the specimens, the protein concentrations were less than 2.3 g/L.

### 3.4. Comparison with Normal Reference Value

**Figure 4:**
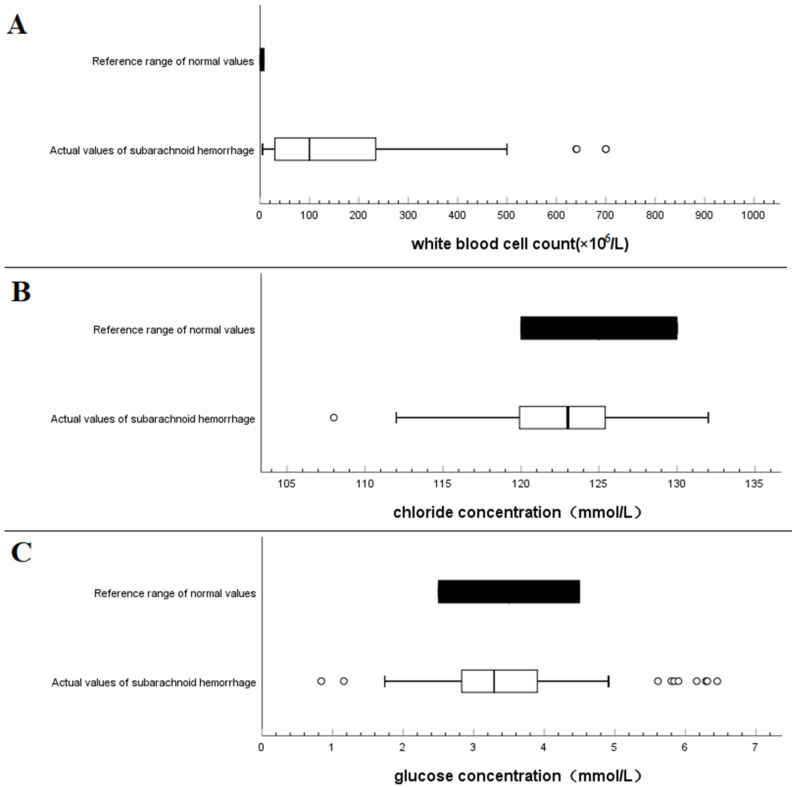
Comparison of the range of variation of cerebrospinal fluid indexes after spontaneous subarachnoid hemorrhage with a normal reference range of cerebrospinal fluid: (**A**) White blood cell count, (**B**) chloride concentration, and (**C**) glucose concentration.

Using boxplots, the lower and upper bound intervals of CSF leukocyte count after spontaneous SAH was determined to be (5–500) × 10^6^ cells/L, with a median value of 100 × 10^6^ cells/L, which was much higher than the normal value range of (0–8) × 10^6^ cells/L (Figure 4A). The lower and upper bound ranges of CSF chloride concentration after spontaneous SAH were found to be (112–132) mmol/L, which was lower than the normal reference value range of (120–130) mmol/L (Figure 4B). Similarly, the lower and upper bound of CSF glucose concentration after spontaneous SAH was observed to be (1.7–4.9) mmol/L, which was lower than the normal reference value range of (2.5–4.5) mmol/L (Figure 4C). These findings indicate significant changes in the CSF indices after spontaneous SAH, particularly in the WBC count, chloride concentration, and glucose concentration when compared with the normal reference values.

## 4. Discussion

The incidence rate of central nervous system (CNS) infections after neurosurgery ranges from 2.5% to 4.6% [1]. CNS infections account for 0.8% to 7% of all CNS infections [2] and have a case fatality rate of 3% to 33% [3]. The typical symptoms and signs of CNS infections include fever, headache, impaired mental consciousness, and cervical tonicity [4,5]. However, these symptoms can also be observed in patients with SAH. Therefore, it can be challenging to distinguish fever due to intracranial infection from that caused by craniotomy for a ruptured cerebral aneurysm.

The routine and biochemical examination of cerebrospinal fluid (CSF) plays a crucial role in the diagnosis and treatment of neurological and neurosurgical diseases, and its reference values are essential for guiding clinical decision-making. However, the CSF results can be significantly altered after aneurysmal subarachnoid hemorrhage (SAH), rendering the normal reference values unreliable. Patients with ruptured cerebral aneurysms often require craniotomy and aneurysm clipping and may experience postoperative symptoms such as fever, headache, cervical rigidity, and impaired consciousness, which could be caused by pulmonary infections, meningeal irritation, or the absorption of bloody CSF. It is challenging for clinicians to differentiate whether these patients have concomitant postoperative intracranial infections based solely on their symptoms. CSF examination is often necessary to aid in diagnosis, but the positive rate of CSF bacterial culture for CNS infection is generally low [6,7]. Moreover, the CSF indexes of patients with SAH are significantly abnormal in this pathological state due to the influence of bloody CSF, making it even more challenging to diagnose intracranial infections after craniotomy. To date, no studies have investigated the fluctuation range of CSF indexes after spontaneous SAH. This study aims to clarify the reference value range of CSF indicators in the pathological condition of spontaneous SAH, which could provide a relative reference value for identifying concomitant CNS infections after craniotomy in such patients.

At present, early diagnosis of CNS infection in neurosurgery still poses a significant challenge [8,9]. The typical CSF manifestations of intracranial infection include a white blood cell count >1000 cells/mm^3^ and a proportion of multinucleated cells >0.7. If the number of leukocytes in CSF is less than 1000 cells/mm^3^, bacterial infection cannot be ruled out. A comprehensive evaluation of risk factors, clinical signs, and other biochemical indicators is necessary to make an accurate diagnosis [10].

According to the literature, a CSF WBC count greater than 100 × 10^6^/L is suggestive of a central nervous system infection [11]. The results of this study show that the median CSF leukocyte count was 100 × 10^6^ cells/L, and the 5–95th percentiles were (8–880) × 10^6^ cells/L. Therefore, 50% of the SAH population would have a CSF WBC count that exceeds the clinical diagnostic criteria for intracranial infection. The median proportion of neutrophils was 46.0%, and 13% of patients had a neutrophil ratio >0.7. The reference standard for CSF routine in current guidelines for the diagnosis of CNS infections is not applicable for judging whether patients with SAH have an intracranial infection. In this study, the CSF WBC count was <880 cells/mm^3,^ and the neutrophil ratio was <0.75 in 95% of the population after spontaneous SAH. Using a “WBC count > 880 cells/mm^3^” as a reference indicator for the presence or absence of intracranial infection would be more instructive in patients with spontaneous SAH. However, bacterial infections cannot be ruled out in patients with white blood cell counts in the range of 100–880 cells/mm^3^. After a subarachnoid hemorrhage (SAH), there is a significant increase in the red blood cell (RBC) count in the cerebrospinal fluid (CSF). This study demonstrates that the 10% to 90% percentile interval for the CSF RBC count in patients with SAH is (900–66,162) × 10^6^ cells/L, while the percentile interval for the ratio of RBC count to white blood cell (WBC) count is (9–1670), which fluctuates widely and is not suitable for clinical use. Despite this, the authors observed that some clinicians still try to convert the RBC/WBC ratio to estimate the actual WBC count in the CSF. However, the authors caution against this method for patients with spontaneous SAH due to two reasons. Firstly, patients are typically bedridden for a prolonged period after the hemorrhage and the RBCs deposit in a low position. Thus, the CSF samples collected through lumbar puncture may not accurately reflect the total RBC content in the CSF. Secondly, the premise of using the RBC count to correct the WBC count is that fresh bleeding occurs during the lumbar puncture procedure. Nonetheless, in the long-term bed rest process for SAH patients, cell separation occurs due to the sedimentation of blood cells, making the conventional correction method of little practical significance.

The normal concentration of glucose in CSF is 2.5–4.5 mmol/L, which is two-fifths of the serum glucose level due to its dependence on serum glucose [11]. In this study, the 5 to 95 percentile interval for CSF glucose concentrations was found to be (2.2–6.3) mmol/L, while the 10 to 90 percentile interval was (2.6–5.5) mmol/L. Furthermore, 8% of the cohort had below-normal CSF glucose concentrations. The reference value for clinical diagnosis of CNS infection in neurosurgery is glucose <2.2 mmol/L [12]. However, the data from this sample indicated that only 5% of the population had CSF glucose concentrations below this value. As such, using “glucose < 2.2 mmol/L” as a reference index for the presence or absence of intracranial infection may be more informative. The normal reference range for CSF chloride concentration is 120–130 mmol/L, which is about 20% higher than the plasma concentration to maintain an osmotic balance between CSF and blood [11]. In pathological conditions, such as septic meningitis, the chloride concentration in CSF can vary and is usually in the range of 102–116 mmol/L [12]. In the SAH population of this sample, the 5% to 95% percentile interval for chloride concentration was (114.9–128.9) mmol/L, with a 10% to 90% percentile interval of (116.8–127.2) mmol/L and a median of 123 mmol/L. The CSF chloride concentration was found to be decreased after SAH, with 5% of the population having a concentration less than 115 mmol/L. Therefore, using “chloride < 115” as a diagnostic index for intracranial infection may be more useful for patients with spontaneous SAH.

Patients with aneurysmal SAH may develop pulmonary infections, which can cause fever and hyperthermia even without invasive neurological treatment. Additionally, most patients will experience symptoms such as impaired consciousness, headache, neck stiffness, and increased intracranial pressure stimulated by bloody CSF. These symptoms can be similar to those of CNS infections, making it difficult to determine whether intracranial infection is present after surgery [13]. Moreover, the culture examination of CSF has a low positive rate and is time-consuming, which is another disadvantage in diagnosing CNS infections [14,15]. To overcome these limitations, researchers have explored alternative methods for the determination of intracranial infections. One such method involves monitoring CSF calcitoninogen [16] and lactate levels [13,17]. However, the cut-off and reference values for these tests are still controversial, and most hospitals have not yet implemented these tests. Magnetic resonance or CT enhancement has also been proposed as an aid in diagnosing CNS infections [18]. However, these tests lack specificity in identifying meningitis [19,20]. Another approach involves macrogenomic examination of pathogens, which identifies pathogen information through genetic sequencing of the sample and comparison with pathogen databases [14]. However, this method is expensive and has a high false-positive rate.

In this study, we conducted a statistical analysis of the pathological changes in cerebrospinal fluid (CSF) indexes following spontaneous subarachnoid hemorrhage (SAH) and established a reference range. Our findings indicate that in 90% of patients with spontaneous SAH, the white blood cell (WBC) count in the CSF was less than 500 × 10^6^/L and less than 880 × 10^6^/L in 95% of patients. The proportion of neutrophils, lymphocytes, and monocytes did not exceed 75%, 75%, and 15%, respectively, in 95% of the population. The red blood cell (RBC) count to WBC count ratio in the CSF exhibited significant variability after SAH. Furthermore, the concentration of chloride, glucose, and protein was >115 mmol/L, >2.2 mmol/L, and <2.3 g/L, respectively, in 95% of the specimens. If the 5–95% quartile interval is taken as the reference range, a “white blood cell count > 880 mm^3^, glucose < 2.2 mmol/L, chloride < 115” can be used as the reference index for detecting the presence of intracranial infection, which may have greater guiding significance for patients with spontaneous SAH. These results are useful for the identification of intracranial infection in patients with SAH, which can reduce the abuse of antibiotics. However, further prospective studies are necessary to validate the reference range.

Our study has several limitations that should be acknowledged. Firstly, it is a retrospective study, and the patients included in the study had severe clinical symptoms and a high degree of SAH. This could have resulted in elevated levels of erythrocytes and leukocytes in the CSF. Future prospective studies are needed to compare CSF indicators in patients with different degrees of hemorrhage. Secondly, the sample size of this study was small. Therefore, the range of CSF index fluctuation in SAH patients needs to be confirmed through larger studies conducted at multiple centers.

## 5. Conclusions

Compared to the normal reference values, the CSF indexes after spontaneous SAH showed significant changes, especially in the leukocyte count, chloride concentration, and glucose concentration. Using “white blood cell count < 880 mm^3^, glucose > 2.2 mmol/L, chloride > 115” as the reference values for SAH pathological status is more meaningful for reference purposes.

## Figures and Tables

**Table 1:** Comparison of demographic and CSF data.

Factors		n (%)	WBC (×10^6^/L)	*p* Value	Chloride (mmol/L)	*p* Value	Glucose (mmol/L)	*p* Value
Gender	Female	67 (66.3)	110 (40–310)	0.038	125.8 (123.4–125.8)	0.059	3.4 (3.0–3.9)	0.274
	Male	34 (33.7)	38 (20–192)		125.1 (122.1–125.1)		3.0 (2.7–3.9)	
Age (years)	<60	61 (60.4)	100 (22–306)	0.532	124 (122.1–125.8)	<0.001	3.3 (2.8–3.9)	0.274
	≥60	40 (39.6)	100 (42–212)		120.7 (117.0–123.7)		3.4 (3.0–4.2)	
treatment method	conservative	15 (14.9)	48 (16–100)	0.04	120.5 (118.0–124.0)	0.089	4.4 (3.6–5.9)	0.002
	endovascular embolization	86 (85.1)	110 (30–310)		123.4 (120.8–125.5)		3.2 (2.8–3.8)	
CSF collection time (days)	≤3	25 (24.8)	120 (35–550)	0.328	125.8 (122.5–127.1)	0.001	3.7 (3.1–4.6)	0.052
	4–7	41 (40.6)	70 (28–153)		123.2 (121.5–125.2)		3.1 (2.8–3.8)	
	>7	35 (34.6)	110 (30–234)		121 (117.0–123.8)		3.3 (2.9–4.0)	

Data are presented as n (%) or median (IQR). IQR indicates interquartile range; CSF, cerebrospinal fluid; and WBC, white blood cell.

**Table 2:** Variation Range of Common CSF Indices after Spontaneous Subarachnoid Hemorrhage.

	RBC (×10^6^/L)	WBC (×10^6^/L)	Leukocyte			Chloride (mmol/L)	Glucose (mmol/L)	Protein (g/L)
			Neutrophil (%)	Monocyte (%)	Lymphocyte (%)			
Average	64,995	241	45.7	9.0	45.7	122.4	3.6	0.9
Standard deviation	215,168	500	19.7	4.0	18.8	4.4	1.2	1.0
*p*-value of normality test	<0.001	<0.001	0.009	<0.001	0.005	0.007	<0.001	<0.001
5th percentile	290	8	10.0	4.6	18.2	114.9	2.2	0.2
10th percentile	900	10	20.0	5.0	20.0	116.8	2.6	0.3
median	19,000	100	46.0	8.0	45.0	123.0	3.3	0.6
90th percentile	66,162	496	70.0	10.0	73.8	127.2	5.5	2.3
95th percentile	355,642	880	75.4	15.0	75.0	128.9	6.3	3.5

RBC indicates red blood cell; and WBC, white blood cell.

## Data Availability

The datasets used and/or analyzed during the current study are available from the corresponding author upon reasonable request.

## Abbreviations

SAH	Subarachnoid hemorrhage
CSF	Cerebrospinal fluid
CNS	Central nervous system
WBC	White blood cell count
RBC	Red blood cell
